# 2-[(*E*)-Benzyl­imino­meth­yl]-4-methyl­phenol

**DOI:** 10.1107/S1600536808010131

**Published:** 2008-04-16

**Authors:** Qi-Feng Liang, Hai-Mei Feng

**Affiliations:** a Department of Chemistry, Jiaying University, Meizhou 514015, People’s Republic of China; b State Key Laboratory Base of Novel Functional Materials and Preparation Science, Institute of Solid Materials Chemistry, Faculty of Materials Science and Chemical Engineering, Ningbo University, Ningbo 315211, People’s Republic of China

## Abstract

In the title Schiff base, C_15_H_15_NO, the benzene rings form a dihedral angle of 74.91 (1)°. There is a strong intra­molecular O—H⋯N hydrogen bond.

## Related literature

For literature on photochromism and thermochromism of Schiff bases in the solid state, see: Cohen *et al.* (1964 ▶).
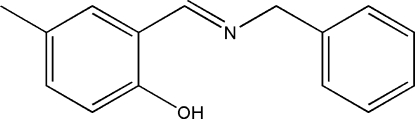


## Experimental

### 

#### Crystal data

C_15_H_15_NO
*M* *_r_* = 225.28
Monoclinic, 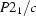

*a* = 14.248 (3) Å
*b* = 6.1724 (2) Å
*c* = 14.529 (3) Å
β = 102.79 (3)°
*V* = 1246.0 (4) Å^3^
*Z* = 4
Mo *K*α radiation
μ = 0.07 mm^−1^
*T* = 295 (2) K
0.54 × 0.30 × 0.25 mm

#### Data collection

Rigaku R-AXIS RAPID IP diffractometer
Absorption correction: multi-scan (*ABSCOR*; Higashi, 1995 ▶) *T*_min_ = 0.970, *T*_max_ = 0.986
11598 measured reflections
2826 independent reflections
1636 reflections with *I* > 2σ(*I*)
*R*_int_ = 0.036

#### Refinement

*R*[*F* ^2^ > 2σ(*F* ^2^)] = 0.049
*wR*(*F* ^2^) = 0.146
*S* = 1.03
2826 reflections
155 parameters
H-atom parameters constrained
Δρ_max_ = 0.15 e Å^−3^
Δρ_min_ = −0.14 e Å^−3^

### 

Data collection: *RAPID-AUTO* (Rigaku, 1998 ▶); cell refinement: *RAPID-AUTO*; data reduction: *CrystalStructure* (Rigaku/MSC, 2002 ▶); program(s) used to solve structure: *SHELXS97* (Sheldrick, 2008 ▶); program(s) used to refine structure: *SHELXL97* (Sheldrick, 2008 ▶); molecular graphics: *CrystalStructure*; software used to prepare material for publication: *SHELXL97*.

## Supplementary Material

Crystal structure: contains datablocks global, I. DOI: 10.1107/S1600536808010131/gk2138sup1.cif

Structure factors: contains datablocks I. DOI: 10.1107/S1600536808010131/gk2138Isup2.hkl

Additional supplementary materials:  crystallographic information; 3D view; checkCIF report

## Figures and Tables

**Table 1:** Hydrogen-bond geometry (Å, °)

*D*—H⋯*A*	*D*—H	H⋯*A*	*D*⋯*A*	*D*—H⋯*A*
O1—H1*A*⋯N1	0.82	1.89	2.616 (2)	147

**Fig. 1.**
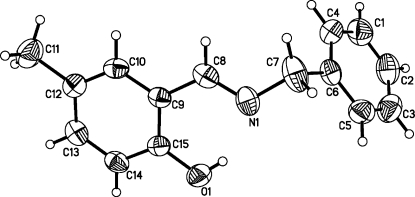
The structure of the title compound, showing 30% probability displacement ellipsoids and the atom-numbering scheme.

## supplementary crystallographic information

### Comment

Compounds presenting photochromism, a reversible color change brought about in at least one direction by the action of electromagnetic radiation, attract considerable attention from various fields of chemistry, physics and material science as potential candidates for practical applications. For a long time, the Schiff bases of salicylaldehyde with aromatic amines (anils or N-salicylideneaniline derivatives) are recognized as such compounds, which undergo keto-enol tautomerism and present common features in their structures and reaction mechanisms (Cohen *et al.*, 1964). The tautomerism involves proton transfer from the hydroxylic oxygen to the imino nitrogen atom that occurs intramolecularly *via* a six-membered ring, with the keto species showing bathochromically shifted spectra. Continuing our studies on the relation between the Schiff base geometry in the crystalline state and photochromism and/or thermochromism, we report here the crystal structure of 2-[(*E*)-(benzylimino)methyl]-4-methylphenol (I).

The molecular structure of (I) is illustrated in Fig. 1. Compound (I) is a typical Schiff base derived from salicylaldehyde with the C8—N1 bond length (Table 1) indicating double-bond character. The title molecule is not planar. The dihedral angle between the phenyl ring and salicylaldimine group is 74.91 (1)°. There is a strong intramolecular hydrogen bond between the phenolic group and the imine N atom (Table 1).

### Experimental

1-Phenylmethanamine (0.02 mol, 2.14 g) and 5-methylsalicylaldehyde (0.02 mol, 2.76 g) were dissolved in ethanol and the solution was refluxed for 3 h. After evaporation, a crude product was recrystallized twice from ethanol to give a pure yellow product. Yield: 87.3%. Calcd. for C_15_H_15_NO: C, 79.97; H, 6.71; N, 6.22; Found: C, 79.53; H, 6.78; N, 6.02%.

### Refinement

All H atoms were located from difference Fourier syntheses. H atoms from the C—H groups and O—H group were placed in geometrically idealized positions and constrained to ride on their parent atoms (C—H = 0.93–0.97%A; O—H = 0.82 Å). and *U*_iso_(H) values equal to 1.2 *U*_eq_(C) or 1.5Ueq(O).

### Crystal data

**Table:**

C_15_H_15_NO	*F*_000_ = 480
*M**_r_* = 225.28	*D*_x_ = 1.201 Mg m^−^^3^
Monoclinic, *P*2_1_/*c*	Mo *K*α radiation λ = 0.71073 Å
Hall symbol: -P 2ybc	Cell parameters from 6530 reflections
*a* = 14.248 (3) Å	θ = 1.0–27.5º
*b* = 6.1724 (2) Å	µ = 0.08 mm^−^^1^
*c* = 14.529 (3) Å	*T* = 295 (2) K
β = 102.79 (3)º	Block, yellow
*V* = 1246.0 (4) Å^3^	0.54 × 0.30 × 0.25 mm
*Z* = 4	

### Data collection

**Table:**

Rigaku R-AXIS RAPID IP diffractometer	2826 independent reflections
Radiation source: fine-focus sealed tube	1636 reflections with *I* > 2σ(*I*)
Monochromator: graphite	*R*_int_ = 0.036
Detector resolution: 0 pixels mm^-1^	θ_max_ = 27.5º
*T* = 295(2) K	θ_min_ = 3.5º
ω scans	*h* = −18→18
Absorption correction: multi-scan(ABSCOR; Higashi, 1995)	*k* = −7→7
*T*_min_ = 0.970, *T*_max_ = 0.986	*l* = −18→18
11598 measured reflections	

### Refinement

**Table:**

Refinement on *F*^2^	Secondary atom site location: difference Fourier map
Least-squares matrix: full	Hydrogen site location: inferred from neighbouring sites
*R*[*F*^2^ > 2σ(*F*^2^)] = 0.049	H-atom parameters constrained
*wR*(*F*^2^) = 0.146	*w* = 1/[σ^2^(*F*_o_^2^) + (0.0657*P*)^2^ + 0.0924*P*] where *P* = (*F*_o_^2^ + 2*F*_c_^2^)/3
*S* = 1.03	(Δ/σ)_max_ < 0.001
2826 reflections	Δρ_max_ = 0.15 e Å^−^^3^
155 parameters	Δρ_min_ = −0.14 e Å^−^^3^
Primary atom site location: structure-invariant direct methods	Extinction correction: none

### Special details

**Table:**

Geometry. All e.s.d.'s (except the e.s.d. in the dihedral angle between two l.s. planes) are estimated using the full covariance matrix. The cell e.s.d.'s are taken into account individually in the estimation of e.s.d.'s in distances, angles and torsion angles; correlations between e.s.d.'s in cell parameters are only used when they are defined by crystal symmetry. An approximate (isotropic) treatment of cell e.s.d.'s is used for estimating e.s.d.'s involving l.s. planes.
Refinement. Refinement of *F*^2^ against ALL reflections. The weighted *R*-factor *wR* and goodness of fit *S* are based on *F*^2^, conventional *R*-factors *R* are based on *F*, with *F* set to zero for negative *F*^2^. The threshold expression of *F*^2^ > σ(*F*^2^) is used only for calculating *R*-factors(gt) *etc*. and is not relevant to the choice of reflections for refinement. *R*-factors based on *F*^2^ are statistically about twice as large as those based on *F*, and *R*- factors based on ALL data will be even larger.

### Fractional atomic coordinates and isotropic or equivalent isotropic displacement parameters (Å^2^)

**Table:**

	*x*	*y*	*z*	*U*_iso_*/*U*_eq_	
O1	0.60971 (8)	−0.30589 (18)	0.62915 (9)	0.0799 (4)	
H1A	0.6539	−0.2326	0.6595	0.120*	
N1	0.69322 (11)	0.0362 (3)	0.71735 (10)	0.0756 (4)	
C1	0.90441 (17)	0.3943 (4)	0.58653 (16)	0.0962 (7)	
H1C	0.9018	0.5185	0.5498	0.115*	
C2	0.96894 (15)	0.2373 (4)	0.58075 (16)	0.0989 (7)	
H2A	1.0104	0.2536	0.5400	0.119*	
C3	0.97336 (16)	0.0579 (4)	0.63369 (17)	0.0958 (7)	
H3A	1.0182	−0.0494	0.6299	0.115*	
C4	0.84281 (13)	0.3717 (3)	0.64613 (14)	0.0820 (5)	
H4A	0.7988	0.4809	0.6495	0.098*	
C5	0.91196 (15)	0.0324 (3)	0.69338 (14)	0.0833 (6)	
H5A	0.9154	−0.0931	0.7294	0.100*	
C6	0.84528 (12)	0.1892 (3)	0.70109 (12)	0.0672 (5)	
C7	0.77651 (14)	0.1640 (4)	0.76503 (14)	0.0978 (7)	
H7A	0.8085	0.0919	0.8228	0.117*	
H7B	0.7554	0.3055	0.7813	0.117*	
C8	0.61136 (13)	0.1263 (3)	0.70132 (11)	0.0661 (5)	
H8A	0.6068	0.2672	0.7225	0.079*	
C9	0.52454 (11)	0.0188 (2)	0.65129 (10)	0.0535 (4)	
C10	0.43697 (12)	0.1266 (2)	0.63498 (11)	0.0616 (4)	
H10A	0.4351	0.2656	0.6592	0.074*	
C11	0.25972 (15)	0.1615 (4)	0.56557 (16)	0.1024 (7)	
H12A	0.2726	0.3131	0.5601	0.154*	
H12B	0.2278	0.1393	0.6165	0.154*	
H12C	0.2192	0.1109	0.5077	0.154*	
C12	0.35323 (12)	0.0375 (3)	0.58492 (11)	0.0657 (4)	
C13	0.35819 (13)	−0.1710 (3)	0.55086 (11)	0.0694 (5)	
H14A	0.3023	−0.2357	0.5167	0.083*	
C14	0.44260 (13)	−0.2846 (3)	0.56589 (12)	0.0671 (5)	
H15A	0.4433	−0.4245	0.5423	0.081*	
C15	0.52682 (11)	−0.1926 (2)	0.61595 (11)	0.0572 (4)	

### Atomic displacement parameters (Å^2^)

**Table:**

	*U*^11^	*U*^22^	*U*^33^	*U*^12^	*U*^13^	*U*^23^
O1	0.0798 (8)	0.0663 (7)	0.1004 (10)	0.0110 (6)	0.0346 (7)	−0.0035 (6)
N1	0.0683 (10)	0.0952 (11)	0.0659 (9)	−0.0153 (8)	0.0206 (8)	−0.0023 (8)
C1	0.0927 (15)	0.0946 (15)	0.1008 (16)	−0.0124 (12)	0.0202 (13)	0.0275 (12)
C2	0.0763 (14)	0.135 (2)	0.0896 (15)	−0.0130 (14)	0.0268 (12)	0.0031 (15)
C3	0.0789 (14)	0.1082 (17)	0.0998 (16)	0.0156 (12)	0.0189 (12)	−0.0135 (14)
C4	0.0696 (12)	0.0780 (12)	0.0946 (14)	0.0086 (9)	0.0096 (10)	0.0031 (11)
C5	0.0920 (14)	0.0717 (11)	0.0777 (13)	−0.0020 (11)	0.0007 (11)	0.0079 (9)
C6	0.0584 (10)	0.0795 (11)	0.0601 (10)	−0.0123 (9)	0.0053 (8)	−0.0041 (8)
C7	0.0822 (13)	0.143 (2)	0.0699 (12)	−0.0370 (13)	0.0208 (10)	−0.0184 (12)
C8	0.0832 (12)	0.0665 (10)	0.0539 (9)	−0.0141 (9)	0.0265 (9)	−0.0049 (8)
C9	0.0675 (10)	0.0496 (8)	0.0470 (8)	−0.0047 (7)	0.0206 (7)	0.0014 (6)
C10	0.0832 (12)	0.0492 (8)	0.0563 (9)	0.0028 (8)	0.0239 (9)	0.0022 (7)
C11	0.0860 (14)	0.1106 (17)	0.1066 (17)	0.0238 (13)	0.0126 (12)	0.0172 (13)
C12	0.0722 (11)	0.0711 (10)	0.0555 (9)	0.0062 (9)	0.0179 (8)	0.0090 (8)
C13	0.0746 (12)	0.0814 (12)	0.0543 (10)	−0.0160 (10)	0.0184 (8)	−0.0064 (8)
C14	0.0848 (12)	0.0565 (9)	0.0675 (10)	−0.0122 (9)	0.0331 (9)	−0.0132 (8)
C15	0.0705 (10)	0.0517 (8)	0.0566 (9)	0.0021 (8)	0.0297 (8)	0.0034 (7)

### Geometric parameters (Å, °)

**Table:**

O1—C15	1.3493 (18)	C7—H7B	0.9700
O1—H1A	0.8200	C8—C9	1.449 (2)
N1—C8	1.266 (2)	C8—H8A	0.9300
N1—C7	1.465 (2)	C9—C10	1.387 (2)
C1—C2	1.351 (3)	C9—C15	1.405 (2)
C1—C4	1.370 (3)	C10—C12	1.368 (2)
C1—H1C	0.9300	C10—H10A	0.9300
C2—C3	1.342 (3)	C11—C12	1.508 (2)
C2—H2A	0.9300	C11—H12A	0.9600
C3—C5	1.370 (3)	C11—H12B	0.9600
C3—H3A	0.9300	C11—H12C	0.9600
C4—C6	1.376 (3)	C12—C13	1.386 (2)
C4—H4A	0.9300	C13—C14	1.367 (2)
C5—C6	1.378 (3)	C13—H14A	0.9300
C5—H5A	0.9300	C14—C15	1.380 (2)
C6—C7	1.500 (2)	C14—H15A	0.9300
C7—H7A	0.9700		
			
C15—O1—H1A	109.5	N1—C8—H8A	118.6
C8—N1—C7	117.85 (18)	C9—C8—H8A	118.6
C2—C1—C4	120.4 (2)	C10—C9—C15	118.39 (15)
C2—C1—H1C	119.8	C10—C9—C8	120.12 (14)
C4—C1—H1C	119.8	C15—C9—C8	121.47 (15)
C3—C2—C1	120.2 (2)	C12—C10—C9	122.94 (15)
C3—C2—H2A	119.9	C12—C10—H10A	118.5
C1—C2—H2A	119.9	C9—C10—H10A	118.5
C2—C3—C5	120.1 (2)	C12—C11—H12A	109.5
C2—C3—H3A	119.9	C12—C11—H12B	109.5
C5—C3—H3A	119.9	H12A—C11—H12B	109.5
C1—C4—C6	120.83 (19)	C12—C11—H12C	109.5
C1—C4—H4A	119.6	H12A—C11—H12C	109.5
C6—C4—H4A	119.6	H12B—C11—H12C	109.5
C3—C5—C6	121.27 (19)	C10—C12—C13	117.10 (16)
C3—C5—H5A	119.4	C10—C12—C11	121.73 (17)
C6—C5—H5A	119.4	C13—C12—C11	121.15 (18)
C4—C6—C5	117.19 (17)	C14—C13—C12	122.12 (16)
C4—C6—C7	120.53 (18)	C14—C13—H14A	118.9
C5—C6—C7	122.28 (18)	C12—C13—H14A	118.9
N1—C7—C6	109.57 (15)	C13—C14—C15	120.28 (15)
N1—C7—H7A	109.8	C13—C14—H15A	119.9
C6—C7—H7A	109.8	C15—C14—H15A	119.9
N1—C7—H7B	109.8	O1—C15—C14	119.53 (14)
C6—C7—H7B	109.8	O1—C15—C9	121.32 (15)
H7A—C7—H7B	108.2	C14—C15—C9	119.15 (15)
N1—C8—C9	122.71 (16)		

### Hydrogen-bond geometry (Å, °)

**Table:**

*D*—H···*A*	*D*—H	H···*A*	*D*···*A*	*D*—H···*A*
O1—H1A···N1	0.82	1.89	2.616 (2)	147
